# Multitemporal single‐cell profiling uncovers alveolar IL1β^hi^ neutrophils: A significant indicator of CARDS progression

**DOI:** 10.1002/ctm2.70479

**Published:** 2025-09-25

**Authors:** Yingying Yang, Haochen Li, Peng Liu, Jinmeng Jia, Lei Wei, Xiangyu Chen, Ziqi Tan, Hantian Li, Qianlin Wang, Siyi Yuan, Liangyu Mi, Yuechuan Xue, Yi Chi, Shuaishuai Yang, Yanjie Zhao, Huaiwu He, Xuegong Zhang, Longxiang Su, Yun Long

**Affiliations:** ^1^ Department of Critical Care Medicine, State Key Laboratory of Complex, Severe, and Rare Diseases Peking Union Medical College Hospital, Peking Union Medical College and Chinese Academy of Medical Sciences Beijing China; ^2^ School of Medicine Tsinghua Medicine, Tsinghua University Beijing China; ^3^ Medical Research Center, State Key Laboratory of Complex, Severe, and Rare Diseases Peking Union Medical College Hospital, Peking Union Medical College and Chinese Academy of Medical Sciences Beijing China; ^4^ MOE Key Lab of Bioinformatics, Bioinformatics Division of BNRIST and Department of Automation Tsinghua University Beijing China; ^5^ 4+4 Medical Doctor Program Peking Union Medical College and Chinese Academy of Medical Sciences Beijing China; ^6^ Department of Toxicology, School of Public Health Qingdao University Qingdao Shandong China; ^7^ Department of Anesthesia, Critical Care and Pain Medicine, Center for Inflammation Research, Beth Israel Deaconess Medical Center Harvard Medical School Boston USA

1

Dear Editor

Acute respiratory distress syndrome (ARDS) is a complex life‐threatening syndrome. Injury mechanisms behind ARDS rapid condition change and their association with treatment efficacy remain unclear. Using multitemporal single‐cell RNA sequencing (scRNA‐seq) on bronchoalveolar lavage fluid (BALF), we provided the first paired human lung immune cell atlas before and after ARDS condition change. We identified IL1β^hi^ neutrophils as dominant in severe ARDS and linked to disease worsening. Neutrophil metabolic reprogramming and interactions with macrophages amplify inflammation, suggesting IL‐1 blockade potential.

This study included eight severe ARDS patients, comprising four COVID‐19‐related ARDS (CARDS) and four non‐CARDS patients (Table S1), with 23 public CARDS samples as controls. BALF samples were obtained upon admission (t1) and condition change (t2; Figure 1A). ScRNA‐seq captured 87044 cells, labelled as major types^1^ (Figure 1B,C). Compared to GSE145926 CARDS BALF data,^1^ our CARDS patients exhibited significantly higher neutrophil proportions. Neutrophils were predominant in our results, followed by macrophages (Figure 1D). ΔMurray scores (MS) categorised patients into exacerbation (ΔMS ≥ +1) and remission groups, confirmed by computed tomography (CT; Figure 1E). In CARDS, after treatment, neutrophils significantly decreased and epithelial cells increased in remission groups, while no such change occurred in exacerbated patients (Figure 1F), suggesting neutrophils as important determinants of CARDS progression. In non‐CARDS, we detected macrophages significantly increase in exacerbated patients, suggesting macrophages may have more impacts on non‐CARDS progression (Figure 1G).

**FIGURE 1:**
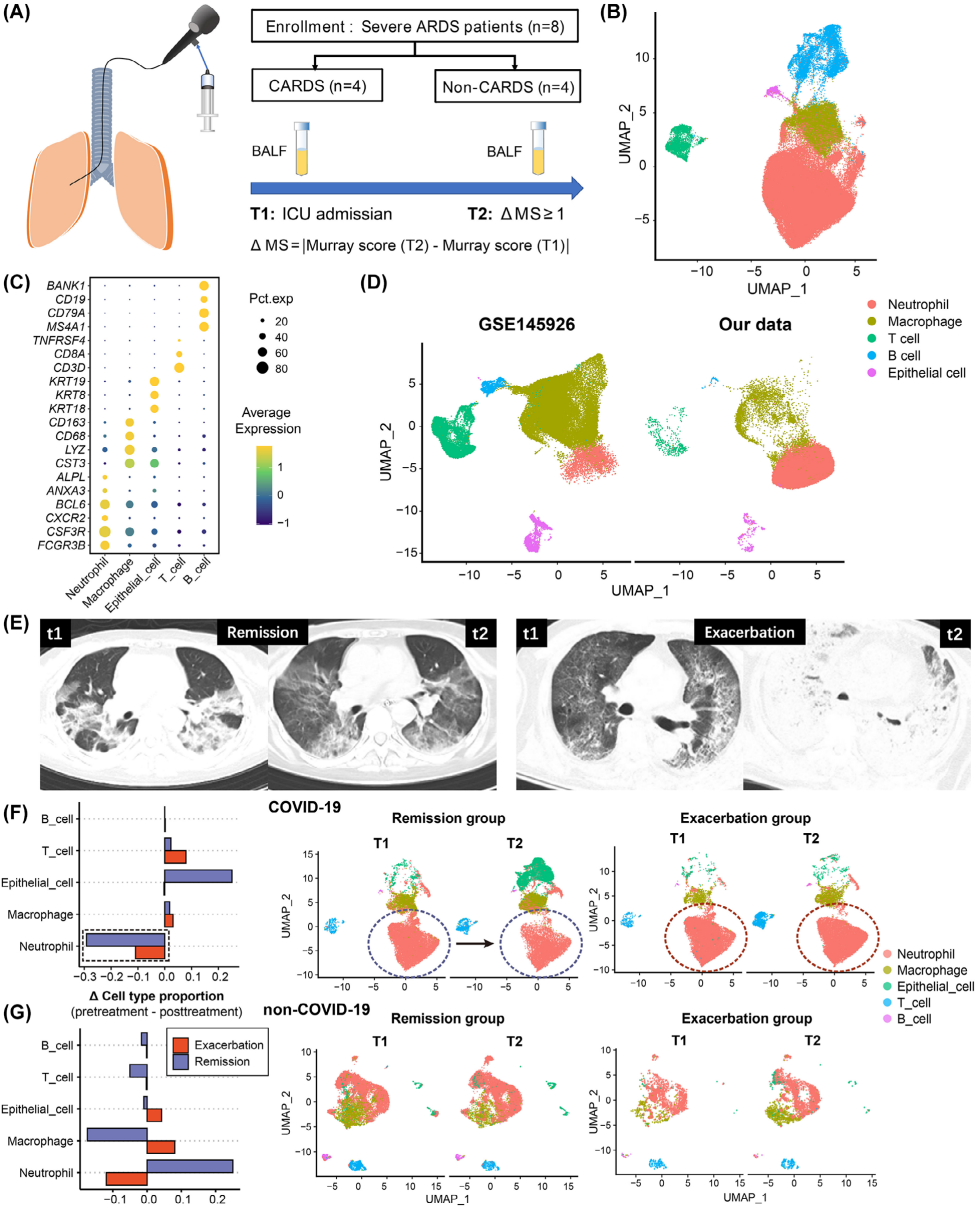
Comparison of the single‐cell profiles of bronchoalveolar lavage fluid (BALF) between exacerbation and remission groups of COVID‐19‐related acute respiratory distress syndrome (CARDS) and non‐CARDS patients. (A) Diagram illustrating patient inclusion and specimen collection process. (B) Uniform manifold approximation and projection (UMAP) visualisation of single cells in BALF from all included patients. (C) Dot plot visualising averaged expression of canonical markers across neutrophils, macrophages, T cells, B cells, and epithelial cells. (D) Comparison of BALF single‐cell profiles between our CARDS patients and GSE145926. The UMAP plot was generated from the integrated analysis of our dataset and GSE145926. (E) Left: Before and after remission in the lung‐injury remission group. Right: Before and after exacerbation in the exacerbation group of lung injury. (F) Comparison of single‐cell profiles (T1 and T2) between the lung‐injury exacerbation group and remission group in CARDS patients. (G) Comparison of single‐cell profiles (T1 and T2) between the lung‐injury exacerbation group and remission group in non‐CARDS patients.

To identify neutrophil subtypes related to ARDS progression, we classified neutrophils into progenitor (CD63+), mature (IL1Β^hi^, S100A12+CXCR2+ and CCL4+), and hybrid neutrophils (CD74+)^2^ (Figures 2A,B and S1A–F). Neutrophil evolution followed two trajectories: immature—S100A12+CXCR2+ neutrophils and immature—IL1β^hi^—S100A12+CXCR2+ neutrophils (Figures 2A and S1G,H). IL1β^hi^ neutrophils significantly increased in exacerbated patients compared to baselines (Figure 2C,D), with upregulated inflammatory genes (*IL1B*, *ISG15*, and *PTGS2*) and interferon‐related pathways (Figure 2E,F). These genes, like *PTGS2*, are identified as key genes in SARS‐CoV‐2 immunopathogenesis.^3^, ^4^ IL1β^hi^ neutrophil signatures and IL1B gene can also be used as prognostic indicators for CARDS (Area under curve [AUC]: .8693, Figure S1I). Comparing our neutrophils with neutrophils from CARDS peripheral blood^5^ (Figure 2G), peripheral neutrophils rarely express IL1β^hi^ neutrophil signatures, while cells with high expression nearly all come from BALF (Figure 2H). This demonstrates that this progression‐related neutrophil group could be unique to BALF, further confirming the uniqueness of our findings.

**FIGURE 2:**
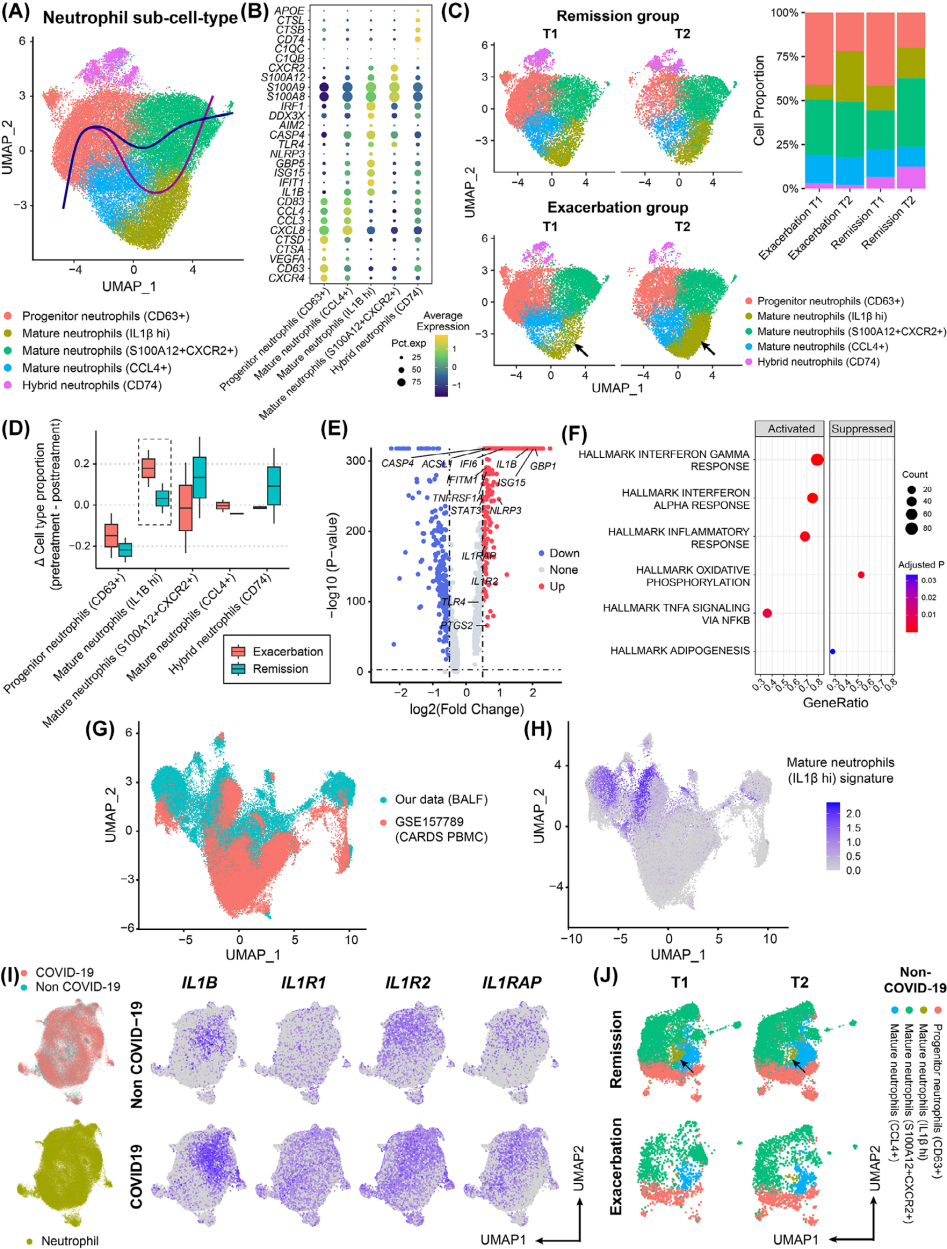
Analysis of neutrophil subtypes in bronchoalveolar lavage fluid (BALF) of COVID‐19‐related acute respiratory distress syndrome (CARDS) and non‐CARDS patients. (A) UMAP of BALF neutrophils in CARDS patients. (B) Dot plot visualising averaged expression of canonical markers across BALF patients. (C) Comparison of single‐cell neutrophil profiles (T1 and T2) between the lung‐injury exacerbation group and remission group in CARDS patients. (D) The difference in the proportion of each neutrophil subtype before and after treatment. (E) Volcano plot showing differentially expressed genes (DEGs) in IL1B+ neutrophils. (F) Gene set enrichment analysis (GSEA) results showing the upregulated and downregulated gene sets of IL1B+ neutrophils. (G) UMAP of the combination of neutrophils in our data and single‐cell data of Sinha et al. (GSE157789) after removing the batch effect. The UMAP plot was generated from the integrated analysis of our neutrophils and the neutrophils from GSE145926. (H) The expression profiles of IL1β^hi^ neutrophil signature in the combined data. (I) Comparing the expression of *IL1B*, *IL1R1*, *IL1R2*, and *IL1RAP* genes in BALF of CARDS and non‐CARDS patients. The UMAP plot was generated from the integrated analysis of neutrophils in CARDS and non‐CARDS patients. (J) Comparison of single‐cell neutrophil profiles (before and after treatment) between the lung‐injury exacerbation group and remission group in non‐CARDS patients.

Comparing CARDS and non‐CARDS, CARDS patients exhibited higher *IL1B*, *IL1R1*, and *IL1RAP* expression than non‐CARDS (Figure 2I), while IL1β^hi^ neutrophils remained low in both non‐CARDS remission and exacerbation groups (Figure 2J). Considering non‐CARDS cell‐type proportions changes (Figure 1F), we think that associations between IL1β^hi^ neutrophils and progression may be unique to CARDS.

Next, we explored pro‐inflammatory mechanisms of IL1β^hi^ neutrophils. Metabolic analysis indicated IL1β^hi^ neutrophils undergo distinct shifts from glucose to fatty acid metabolism (Figure 3A,B), with elevated lipid‐metabolism enzymes (e.g., ACSL1; Figure 3C). Studies have shown the relationship of ACSL1 with IL1β release and neutrophil chemotaxis.^6^ Transcription factor (TF) analysis revealed upregulation of NFKB2, RELB, and STAT (Figure 3D). Thus, we hypothesise that elevated neutrophil ACSL1 may contribute to lysosomal stress via intracellular lipid accumulation, which could activate inflammatory pathways and facilitate IL1β release. Also, elevated ACSL1 promoted fatty acid oxidation, increased mitochondrial metabolism and ATP production, which also promoted neutrophil differentiation (Figure 3E).

**FIGURE 3:**
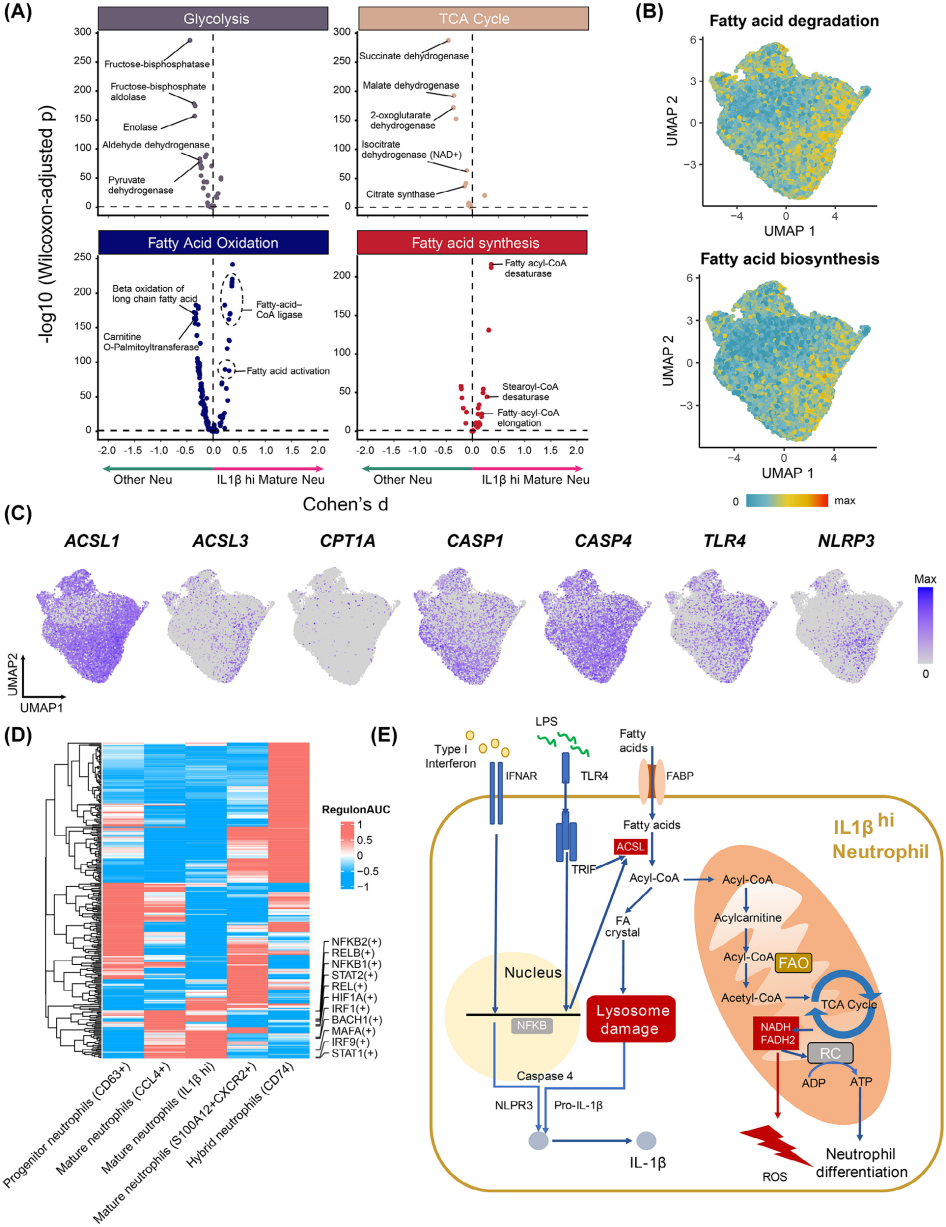
Metabolic and regulatory network analysis on IL1β^hi^ neutrophils. (A) Compass‐score differential activity test results comparing IL1B+ neutrophils and other types of neutrophils. (B) Quantifying metabolism activity at the single‐cell resolution using the ‘scMetabolism’ package. (C) Expression of *ACSL1*, *ACSL3*, *CPT1A*, *CASP1*, *CASP4*, *TLR4* and *NLRP3* in IL1B+ neutrophils. (D) Heatmap of regulon activity across five neutrophil groups, showing the active transcription factors of IL1B+ neutrophils. (E) Schematic diagram of the hypothesised mechanism in IL1β^hi^ neutrophils for receiving extracellular signals, undergoing genetic regulatory changes, releasing IL1β and metabolic state changing.

To investigate relationships between other cell types and IL1β^hi^ neutrophils, we classified macrophages into subclusters (Figure 4A,B). Highly inflammatory M1 macrophages (IFIT+CXCL10+) increased in exacerbated patients, suggesting their association with CARDS progression (Figures 4C,D and S2A), with upregulation of interferon‐related genes (*IFIT3*, *ISG20*, *ISG15*) and located at the end of pseudotime trajectories as a pathogen‐responsive state (Figures 4E,F and S2B,C). Fibrosis signatures, which significantly upregulated in immunomodulatory profibrotic M2 and alveolar macrophages, aren't enriched in IFIT+CXCL10+ macrophages (Figure S2D). Metabolism analysis further detected increased oxidative phosphorylation in IFIT+CXCL10+ macrophages (Figure S2E). While prior studies focused on macrophage‐derived IL1β,^7^, ^8^ our findings suggest IL1β‐related neutrophils may have stronger associations with progression.

**FIGURE 4:**
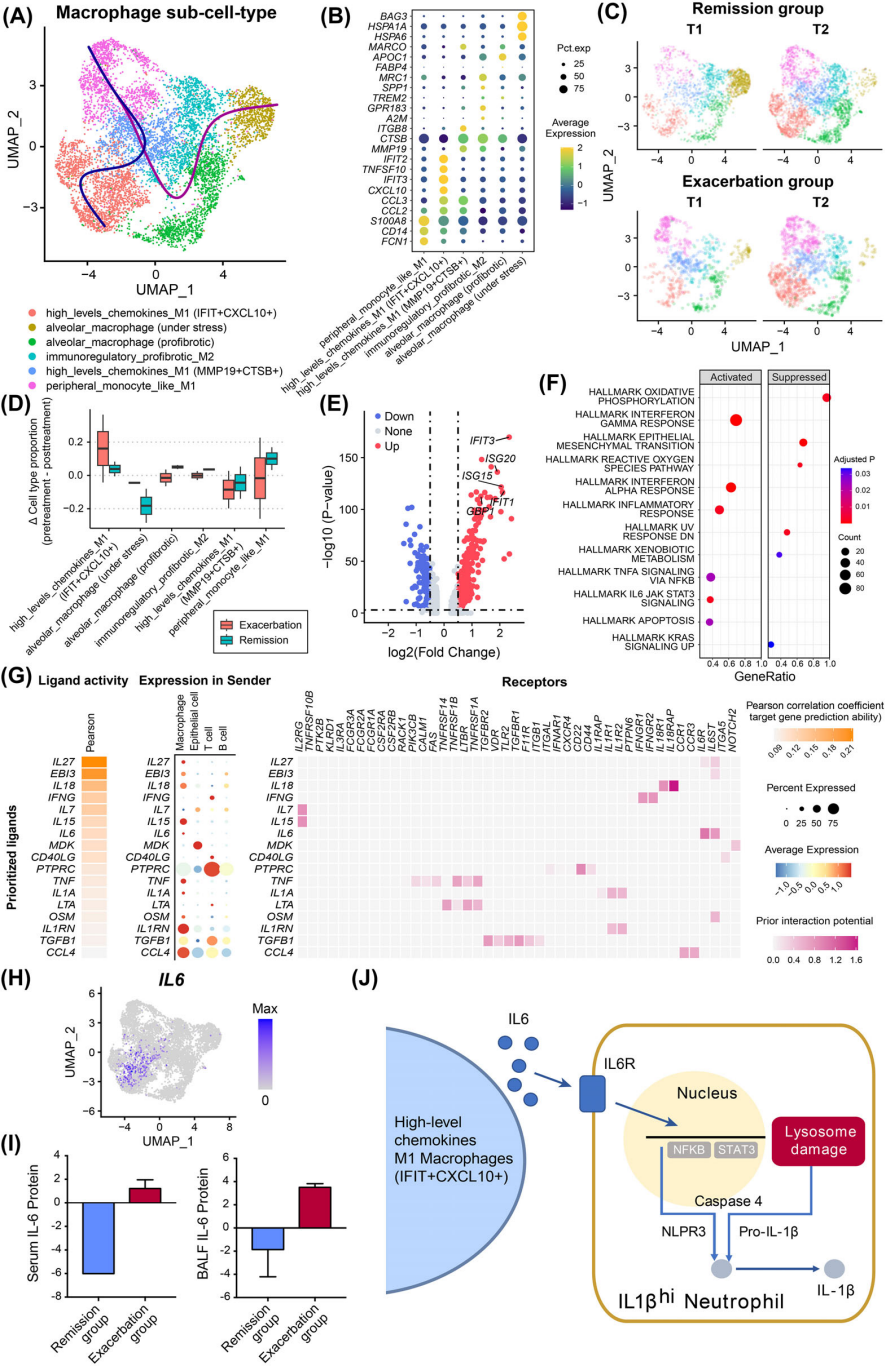
Analysis of bronchoalveolar lavage fluid (BALF) macrophage subtypes in COVID‐19‐related acute respiratory distress syndrome (CARDS) patients. (A) BALF macrophage subtypes in CARDS patients. (B) Annotation of macrophage cell types. (C) Comparing the macrophage subtype change induced by treatment in CARDS remission and exacerbation groups. (D) The difference in the proportion of each macrophage subtype before and after treatment. (E) Differential gene expression analysis of highly inflammatory M1 macrophages (IFIT+CXCL10+). (F) The upregulation and downregulation pathways of highly inflammatory M1 macrophages (IFIT+CXCL10+). (G) Results of NicheNet's ligand activity prediction on differentially expressed genes (DEGs) of IL1β^hi^ neutrophils. Heatmaps show the ranked prioritised ligands, the sender cell types and the corresponding receptors of these ligands. (H) The expression profiles of *TNF* and *CCL4*. (I) The change of IL‐6 protein levels in peripheral blood and BALF from patients in the exacerbation and remission groups. (J) The hypothesised mechanism of IFIT+CXCL10+ M1 macrophage inducing the pro‐inflammatory phenotype of IL1β^hi^ neutrophils.

Our analysis revealed strong inflammatory macrophage–neutrophil interactions, with macrophage‐derived ligands (particularly IL‐6 from IFIT+CXCL10+ macrophages) prominently activating IL1β^hi^ neutrophils (Figure 4G,H). Olink confirmed IL‐6 and other proteins related to neutrophil activation were significantly elevated in BALF supernatant and peripheral blood plasma of exacerbated patients (Figures 4I and S2F). Studies have shown that neutrophils can activate STAT3 and NFKB under IL6, thereby mediating high intracellular inflammation.^9^ Our analysis also suggests these two TFs activate in IL1β^hi^ neutrophils. Confirming IL1B and IL6 cellular sources, we speculate that IFIT+CXCL10+ macrophages act on neutrophils by releasing IL6, causing high inflammation (Figures 4J and S4G,H).

Also, considering IL1β^hi^ neutrophils as upstream cells, we found that IL1β/TNF from IL1β^hi^ neutrophils may potentially induce IFIT+CXCL10+ macrophages (Figure S2I,J). IL1β receptors are highly expressed in IFIT+CXCL10+ macrophages (Figure S2J), further suggesting pro‐inflammatory positive feedback loops with IL1β^hi^ neutrophils and IFIT+CXCL10+ macrophages, causing lung‐injury exacerbation.

As all these patients had baseline immunosuppression, we investigated neutrophil–T interactions. Identifying T‐cell subgroups (Figure S3A,B) and comparing IL1β^hi^ neutrophil–T and other neutrophil–T interactions, we found IL1β^hi^ neutrophil–T with stronger PDL1/PD1 signalling (Figure S3C). We further confirmed PDL1 mainly expressed in IL1β^hi^ neutrophils and PD1 mainly in Treg (Figure S3D). These indicate that in immunosuppressed CARDS patients, neutrophil‐induced immunosuppression potentially accelerates cellular immune decline, warranting further attention.

In conclusion, we provided the first multitemporal human ARDS lung immune cell atlas, to our knowledge. Neutrophils dominate BALF samples, with alveolar‐specific IL1β^hi^ neutrophils increasing during exacerbation, suggesting links to ARDS progression. These neutrophils express inflammation‐related genes and exhibit metabolic signatures suggestive of shifts from glucose to fatty acid metabolism, potentially associated with pro‐inflammatory phenotypes. Bioinformatic analysis indicated they may also act as intercellular communication hubs, forming signalling axes with macrophages and T cells. BALF proteomics confirmed the IL‐6 signals. IL‐1 blockers are found with ability to control inflammation in various diseases, which effect on ARDS also was supported by retrospective cohort studies and mouse models.^10^ Our findings highlight IL‐1 signalling and alveolar inflammatory loops as potential therapeutic targets in severe ARDS, although further validation is required. While our single‐cell transcriptomic data suggest associations between alveolar immune changes and CARDS progression, direct experimental validation remains necessary. Future experiments are warranted to assess whether IL‐6 or fatty acid exposure can induce IL1β^hi^ neutrophil phenotypes in vitro, and evaluate the therapeutic impact of neutrophil depletion, IL‐1 blockade, or metabolic inhibition in murine ARDS models.

## AUTHOR CONTRIBUTIONS

Yingying Yang, Haochen Li, Peng Liu, Jinmeng Jia, Lei Wei, Xiangyu Chen, Ziqi Tan, Hantian Li, Qianlin Wang, Siyi Yuan, Liangyu Mi, Yuechuan Xue, Yi Chi, Shuaishuai Yang, Yanjie Zhao: Substantial contributions to the conception or design of the work; or the acquisition, analysis, or interpretation of data for the work; Yingying Yang, Haochen Li, Peng Liu, Jinmeng Jia, Lei Wei, Longxiang Su, Yun Long: Drafting the work or reviewing it critically for important intellectual content; Longxiang Su, Yun Long: Final approval of the version to be published; Longxiang Su, Yun Long: Agreement to be accountable for all aspects of the work.

## CONFLICT OF INTEREST STATEMENT

The authors declare no conflicts of interest.

## ETHICS STATEMENT

All patients signed an informed consent for study enrolment. The study was supported by the Ethics Committee of Peking Union Medical College Hospital (ZS‐3391).

## Supporting information



Supporting Information

Supporting Information

Supporting Information

## Data Availability

The accession number for the raw and processed sequencing data reported in this paper is Gene Expression Omnibus (GEO): GSE240580. Public data were also collected from GSE145926, GSE157789, and GSE136831.
