# Supplementary material for: Multitemporal single‐cell profiling uncovers alveolar IL1βhi neutrophils: A significant indicator of CARDS progression
Source: Clin Transl Med. 2025 Sep 25;15(10):e70479. doi: 10.1002/ctm2.70479 (PMC12463734; doi:10.1002/ctm2.70479)
Supplement: Supplementary file 1 — Supporting Information [file CTM2-15-e70479-s001.docx]

# **Supplementary figures**


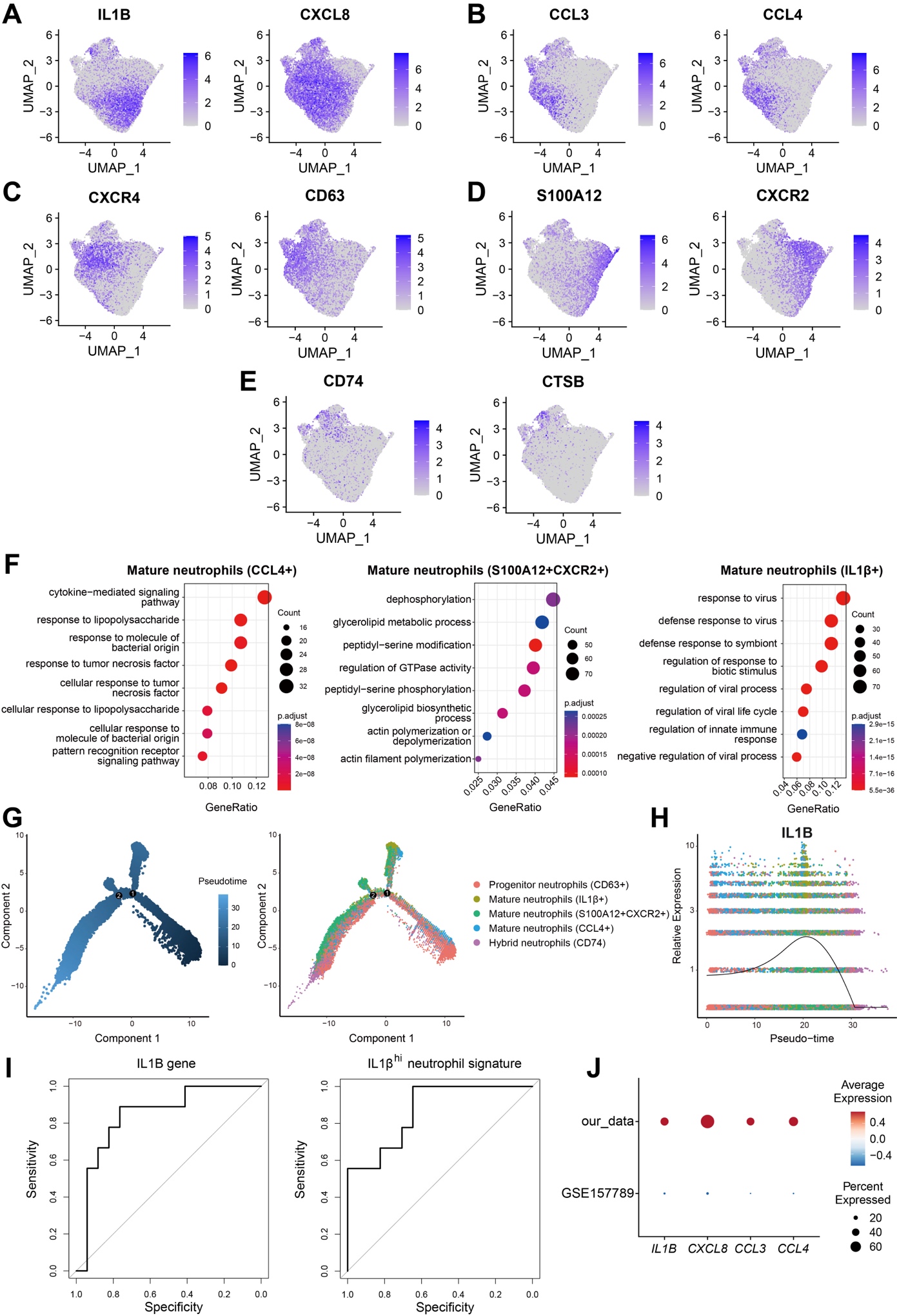


**Figure S1. The expression profiles of the main marker genes in each neutrophil subtype.**

**(A-E)** Expression of marker genes of each neutrophil subtype. **(F)** GO enrichment of neutrophil subtypes. **(G)** Pseudotime analysis of neutrophils and the distribution of neutrophil subtypes along with pseudotime. **(H)** The expression changes of IL1B along with pseudotime in neutrophils. **(I)** ROC curve for IL1B gene and IL1β^hi^ neutrophil signature as prognostic indicator for CARDS. **(J)** Dot plot for comparing IL1β^hi^ neutrophil marker genes between our BALF single-cell data and GSE157789 PBMC data.


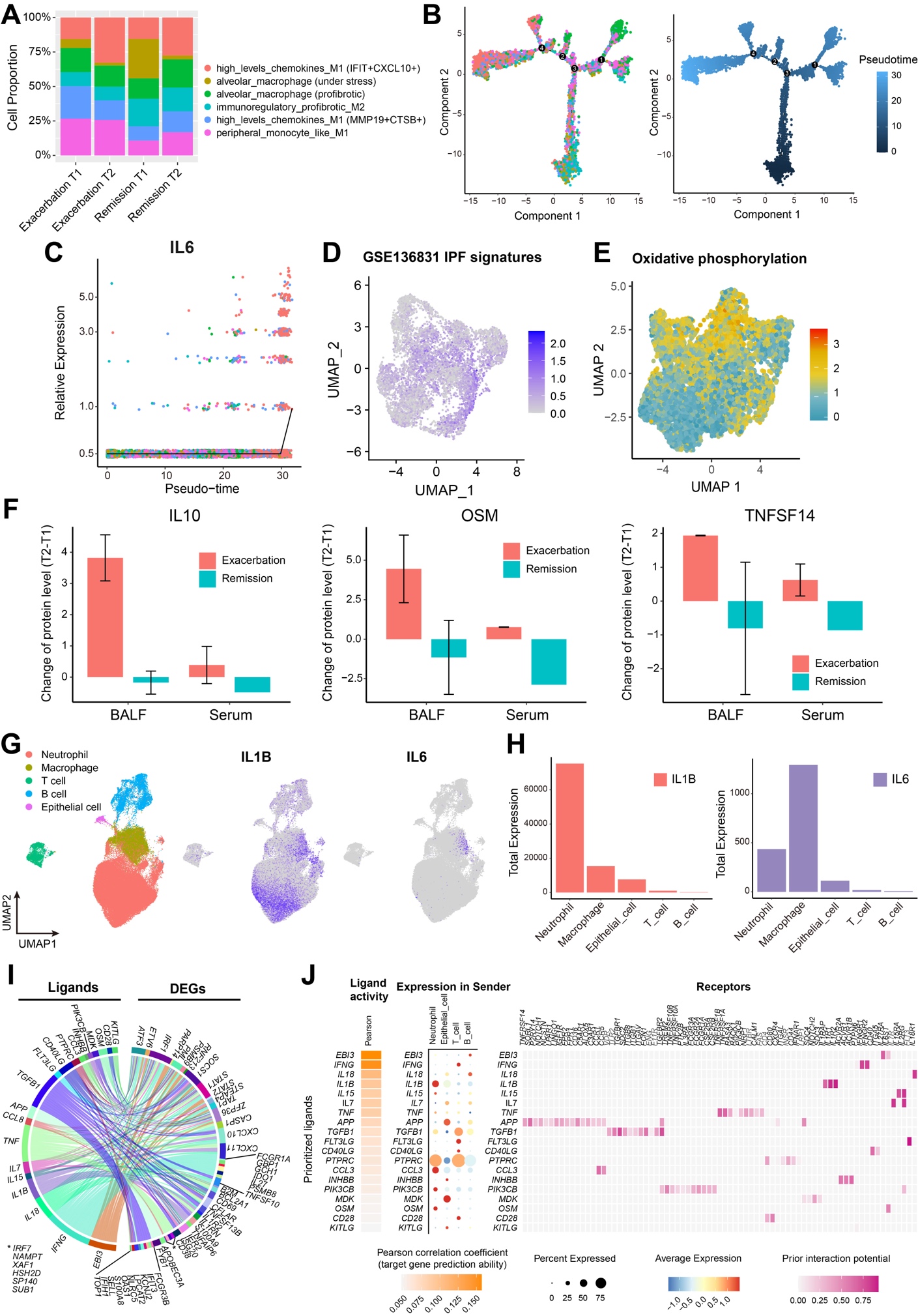


**Figure S2. Analysis on BALF macrophages and their communication with neutrophils.**

**(A)** The proportion of macrophage subtypes in each group and time point. **(B)** Pseudotime analysis of macrophages and the distribution of macrophage subtypes along with pseudotime. **(C)** The expression changes of IL6 along with pseudotime in macrophages. **(D)** The profile of IPF signature in macrophage. **(E)** Quantifying metabolism activity of macrophages at the single-cell resolution. **(F)** The change of IL-10, OSM, and TNFSF14 protein levels in peripheral blood and BALF from patients in the exacerbation and remission groups. **(G)** The expression of IL1B and IL6 in all cell types. **(H)** Cellular sources of IL1B and IL6. **(I)** Circos plot showing arrows between DEGs of IFIT+CXCL10+ M1 macrophages and ligands. One Arrow represents that the ligand could regulate the DEG. **(J)** NicheNet analysis of IL1β+ neutrophils acting on highly inflammatory M1 macrophages (IFIT+CXCL10+).


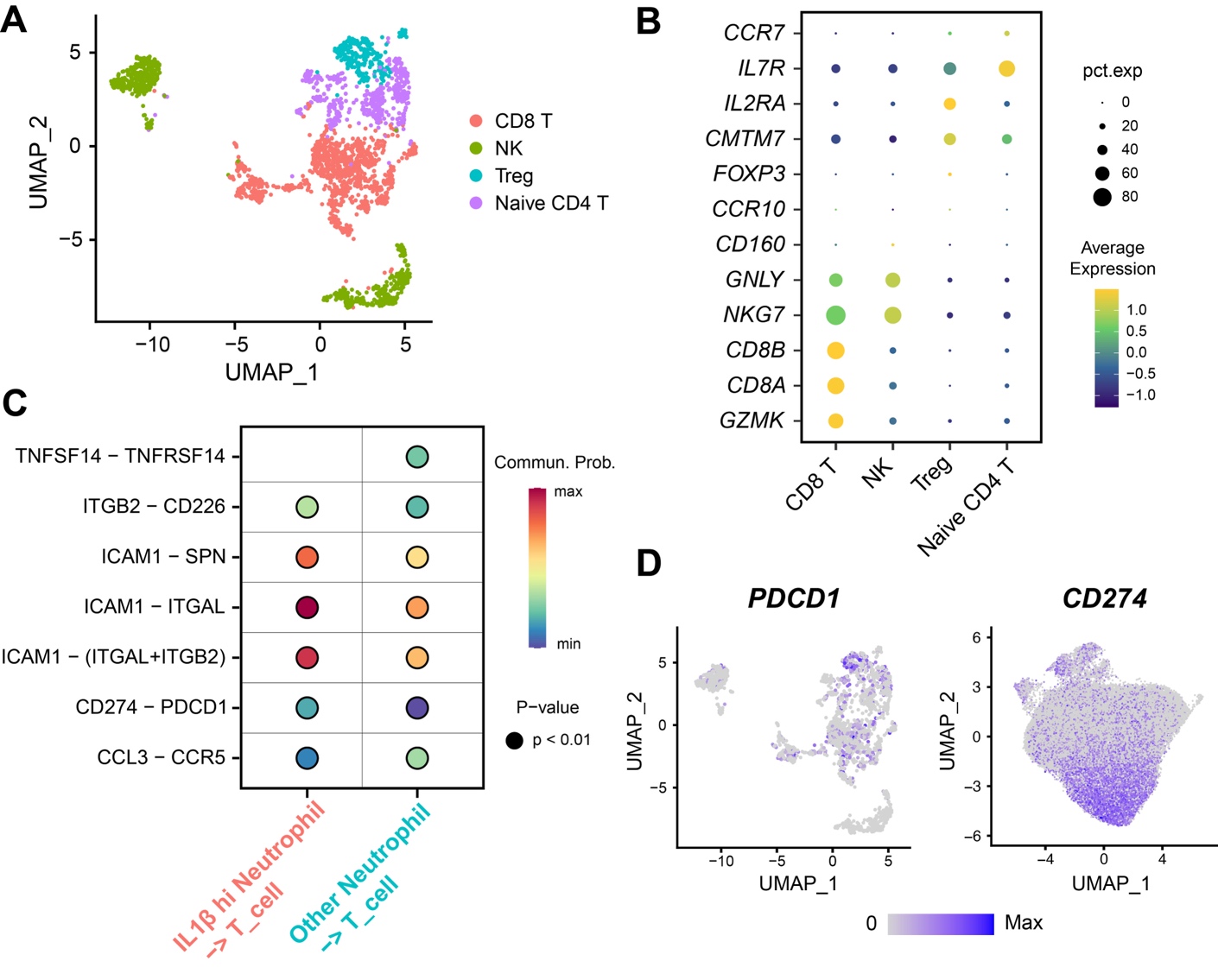


**Figure S3. Analysis of T cells subtypes and their interactions between neutrophils in CARDS patients.**

**(A)** UMAP of BALF T cells in CARDS patients. **(B)** Dot plot visualizing averaged expression of canonical markers across BALF patients. **(C)** Cellchat intercellular communication analysis shows that IL1β+ neutrophils and other neutrophils have different patterns of interaction with T cells. Dot plot shows the ligand–receptor pairs with different interaction strengths **(D)** The expression profiles of *PDCD1* (encoding PD1) in T cells and *CD274* (encoding PDL1) in neutrophils.
