# Supplementary material for: Multitemporal single‐cell profiling uncovers alveolar IL1βhi neutrophils: A significant indicator of CARDS progression
Source: Clin Transl Med. 2025 Sep 25;15(10):e70479. doi: 10.1002/ctm2.70479 (PMC12463734; doi:10.1002/ctm2.70479)
Supplement: Supplementary file 2 — Supporting Information [file CTM2-15-e70479-s002.docx]

**Table S1. Clinical information**

|  | CARDS (n=4) | | | | Non-CARDS (n=4) | | | |
| --- | --- | --- | --- | --- | --- | --- | --- | --- |
|  | C1(FZH） | C2(XZZ) | C3(XQ) | C4(QCX) | N1(LML) | N2(RZQ) | N3(CXM) | N4(WYQ) |
| Age (years) | 78 | 79 | 54 | 66 | 66 | 61 | 65 | 55 |
| Sex | M | M | F | M | F | M | M | F |
| APACHE II | 22 | 20 | 26 | 35 | 29 | 28 | 26 | 18 |
| SOFA | 13 | 12 | 12 | 14 | 11 | 15 | 12 | 10 |
| Lymphocyte count (*10^9^/L) (0.8-4.0) | 0.36 | 0.70 | 0.08 | 0.25 | 0.59 | 0.46 | 0.52 | 0.27 |
| Bacterial pneumonia | - | - | - | - | Y | Y | N | N |
| Fungal pneumonia | - | - | - | - | N | N | Y | Y |
| Murray Score (t1) | 3 | 2 | 2 | 3.75 | 2.25 | 2.75 | 2.5 | 2.5 |
| FiO2/PaO2 (mmHg) | 60 | 149 | 219 | 100 | 180 | 150 | 180 | 190 |
| Daily maximum dose of steroid (mg) | 80 | 80 | 80 | 160 | NA | 320 | 80 | 80 |
| Prone position | Y | Y | Y | Y | Y | Y | Y | Y |
| ECMO | Y | N | N | Y | N | N | N | N |
| Murray Score (t2) | 4 | 3 | 1 | 2 | 1 | 3.75 | 1 | 1 |
| Murray Score (t2) - Murray Score (t1) | 1 | 1 | -1 | -1.75 | -1.25 | 1 | -1.5 | -1.5 |
| Remission Status (t2) | N | N | Y | Y | Y | N | Y | Y |
| Mechanical ventilation (hours) | 2399 | 573 | 223 | 1200 | 157 | 180 | 1200 | 43 |
| Length of ICU stay (days) | 125 | 39 | 14 | 60 | 16 | 18 | 103 | 6 |

**Abbreviations:** “M”=Male,“F”=Female, “APACHEII”=Acute Physiology and Chronic Health Evaluation II, “SOFA”=Sequential Organ Failure Assessment, “ECMO”=Extracorporeal Membrane Oxygenation, “Y”= YES, “N”= No, “NA” = Not Applicable
