# Supplementary material for: Multitemporal single‐cell profiling uncovers alveolar IL1βhi neutrophils: A significant indicator of CARDS progression
Source: Clin Transl Med. 2025 Sep 25;15(10):e70479. doi: 10.1002/ctm2.70479 (PMC12463734; doi:10.1002/ctm2.70479)
Supplement: Supplementary file 3 — Supporting Information [file CTM2-15-e70479-s003.docx]

# Supplementary Methods

## Patient Enrollment

This is a prospective study (NCT05933291), which included mechanically ventilated patients who met the diagnostic criteria for ARDS and COVID-19 infection. Enrolled ARDS patients underwent lung CT at baseline and a second CT with an absolute change in Murray Score (MS) of >1 point. MS is a comprehensive 4-factor scoring system, including hypoxemia (PaO2/FiO2), PEEP (positive end-expiratory pressure), lung compliance, and imaging (CXR quadrigrams infused), and its changes are often used to reflect the progression of lung injury ^1^. The condition of patients at each time point was also confirmed by CT (**Figure 1E**).

All patients signed an informed consent for study enrollment. The study was supported by the Ethics Committee of Peking Union Medical College Hospital (ZS-3391).

## Sample collection

Following the guidelines of the Ethics Committee of Peking Union Medical College Hospital, the patient in this case has signed the informed consent. The collection of BALF followed the recommended bronchoscopy procedure. Once the patient was sedated and received analgesia, 0.9% saline was injected into either the middle lobe of the right lung or the upper lobe of the left lung to obtain at least 20 mL of BALF from each patient. The freshly collected BALF was promptly subjected to scRNA-seq analysis.

## Isolation of BALF cells

The BALF was passed through a 70µm nylon cell strainer (ThermoFisher Scientific, 22-363-548) to remove clumps and debris. The cells were then centrifuged at 300 g for 5 min at 4℃ (LICHEN, LC-LX-LR55C), and the supernatant was discarded. The cells were re-suspended in PBS (Bioss, C7033) containing 0.04% BSA (Solarbio, s9020) and counted using a Countstar Rigel S2 instrument (Shanghai Ruiyu Biotechnology). We adjusted the cell suspension concentration to 300-600 living cells per microlite.

## Single-Cell RNA Sequencing

Single-cell capturing and downstream library construction were performed using the Chromium Next GEM Single Cell 3'GEM Kit v3.1 (10x Genomics), following the manufacturer's protocol. In brief, a total of 70 µL of Master Mix and Cell Suspension (containing 6,000 cells), 50 µL of barcoded gel beads, and 45 µL of partitioning oil were loaded onto Chromium Chip K to generate single-cell gel bead-in-emulsion (GEM). Immediately following GEM generation, the gel beads were dissolved, and primers were released. The polyadenylated mRNA was reverse-transcribed into cDNA. Full-length cDNA, along with cell barcode identifiers, was PCR-amplified, and sequencing libraries were prepared and normalized. The libraries were finally sequenced using an Illumina Novaseq6000 sequencer, with a sequencing depth of at least 100,000 reads per cell, using the paired-end 150 bp (PE150) reading strategy (Bestopcell, Beijing).

## Data preprocessing and quality control

The Cell Ranger software was utilized to perform alignment, filtering, barcode counting, and Unique Molecular Identifier (UMI) counting to create a feature-barcode matrix (<https://support.10xgenomics.com/single-cell-gene-expression/software/downloads/latest)>. To eliminate cells with poor sequencing quality, we established specific criteria for each cell, including a UMI count between 500 and 20000, a gene number greater than 300, and a mitochondrial gene percentage less than 10%. After filtering, we loaded the resulting gene-barcode matrix into Seurat v4 and normalized it using the ‘LogNormalize’ method. We then utilized the ‘vst’ method to identify the top 2,000 variable genes and regressed out the differences in UMI counts and mitochondrial gene percentage of cells using the “ScaleData” function. Then we performed principal component analysis (PCA) using these genes. These processes were performed using default parameters in Seurat v4. Harmony was then used to address batch effects across samples ^2^. Then we performed uniform manifold approximation and projection (UMAP) and graph-based clustering analysis on the top 30 components obtained by Harmony using functions in Seurat v4.

## Public data collection

For public CARDS samples, 9 samples with BALF single-cell data were collected from GSE145926 ^3^ and 14 samples with blood single-cell data were collected from GSE157789 ^4^. We also collected 78 public samples from IPF, COPD and control lungs in GSE136831 ^5^ to calculate IPF signature.

## Cell type annotation

We performed unsupervised clustering to partition the cells into clusters. The resolution parameter in the clustering function was set to 1.2. Then, we assigned clusters into known cell-types using following marker gene sets ^3,6^: Neutrophils: *FCGR3B*, *CSF3R*, *CXCR2*, *BCL6*, *ANXA3*, *ALPL*; Macrophage: *CST3*, *LYZ*, *CD68*, *CD163*; T cells: *CD3D*, *CD8A*, *TNFRSF4*; Epithelial cells: *KRT18*, *KRT8*, *KRT19*; B cells: *MS4A1*, *IGHG4*, *CD19*, *BANK1*. Subsequently, to precisely delineate the boundary between neutrophils and macrophages, we performed separate re-clustering on both cell populations, enabling refined classification of a small subset of ambiguously assigned cells. Then we selected neutrophils and re-clustered them. The resolution parameter in the clustering function was set to 1.2 to obtain a finer result.

When analyzing the subtype of some specific cell-types like neutrophils, we extracted the specific cell type from all cells and we re-performed steps including “NormalizeData”, “FindVariableFeatures”, “ScaleData”, “RunPCA”, “RunHarmony”, and clustering, with the same parameters selected as described before.

We identified the sub-clusters of neutrophils using the following marker gene sets ^7^: Inflammatory mature neutrophils (IL1β+) with high-level cytokines and chemokines: *IL1B*, *CXCL8*; Inflammatory mature neutrophils (CCL4+) with high-level cytokines and chemokines: *CCL3*, *CCL4*, *CXCL8*; Progenitor neutrophils (CD63+): *CXCR4*, *CD63*, *VEGFA*, *CTSA*, *CTSD*; Inflammatory mature neutrophils (S100A12+), with IFN-induced genes and calgranulins: *S100A8*, *S100A9*, *S100A12*, *CXCR2*; Hybrid neutrophils (CD74+), with macrophage-like characteristics: *C1QB*, *C1QC*, *CD74*, *CTSB*, *CTSL*, *APOE*.

We identified the sub-clusters of macrophages using the following marker gene sets ^3^: Peripheral monocyte like M1: *FCN1*, *CD14*, *S100A8*; High levels chemokines M1: *CCL2*, *CCL3*, *CXCL10*; Immunoregulatory profibrotic M2: *A2M*, *GPR183*, *TREM2*, *SPP1*, *CD163*, *MRC1*; Alveolar macrophage: *FABP4*, *APOC1*, *MARCO*.

We also confirmed the cell type annotation results by the differentially expressed genes (DEGs). DEGs are detected by the “FindMarkers” function, and we defined DEGs as the genes with adjusted p-value < 0.001 and the logarithm of fold change (logFC) > 0.5.

## Gene set enrichment analysis

Gene set enrichment analysis (GSEA) ^8^ was performed on the DEGs of the specific cell type of interest using the ‘clusterProfiler’ package. The analysis was based on the Hallmark molecular signatures database ^9^. The enriched gene sets with p-value<0.05 were divided into activated and suppressed gene sets, and plotted in a dot plot using the ‘enrichplot’ package.

## Metabolism analysis

We performed Compass, an algorithm characterizing the metabolic state of each single cell, on the single-cell data of neutrophils. We followed the workflow of Wagner et al ^10^ for the parameter selection and analyzing pipeline. The reaction metadata are derived from the Recon2 database ^11^. Compass-score differential activity analysis was conducted based on the Wilcoxon rank sum test and Cohen’s d statistic on the Compass score of each reaction. Also, we used the “sc.metabolism.Seurat” function with the default parameters in the “scMetabolism” package ^12^ to quantify metabolism activity at the single-cell resolution and verify the results of Compass.

## Gene regulatory network analysis

We inferred the single-cell regulatory network using pySCENIC ^13^. GRNBoost2 algorithm was used to construct gene regulatory networks. Then we selected potential direct-binding targets (regulons) and calculated regulon activity (AUC) scores for each single cell. Then we averaged RegulonAUC scores for each neutrophil cluster and scaled these scores. The analyzing results are plotted using the ‘ComplexHeatmap’ package.

## Ligand-receptor interaction analysis

Currently, there are more and more computational methods that can reveal intercellular communication through omics data ^14–18^. We inferred ligand-target links in single-cell data during remission using the ‘nichnetr’ package ^15^. We regarded all cell types as sender cell types, neutrophils as receiver cell types, and genes expressed in more than 5% of cells as background genes. We performed differential expression analysis and selected the DEGs (adjusted p-value≤0.001 and average log2FC≥0.5) of the selected receiver cell type as the gene set of interest. NicheNet provides the prioritized ligands related to the DEGs of the selected receiver cell type, indicating these ligands could lead to the special cellular phenotype of the selected receiver cell type. The ligand-target links are provided by the ‘get_weighted_ligand_target_links’ function in the ‘nichnetr’ package, and are plotted in circos plot using the ‘circlize’ package.

Also, we compared the cellular communication networks formed by IL1β+ neutrophils and other neutrophils with other cell types using Cellchat ^16^. We divided neutrophils into IL1β+ neutrophils and other neutrophils, and constructed cell communication networks with other cell types, respectively. We used the computeCommunProb function with 'truncatedMean' methods and set the trim parameter as 0.2. Then, we merged the two cellchat objects including different types of neutrophils by the mergeCellChat function. And we used the netVisual_bubble function to compare different interactions, and selected ligand–receptor pairs with a communication strength difference greater than 0.03 to visualize in the dot plot.

## Cell trajectory analysis

﻿We used Slingshot ^19^ for inferring pseudo-time trajectories and aligning cells to the trajectories. Slingshot is a package constructing a ﻿minimal spanning tree (MST) on clusters of cells and ﻿identifying pseudo times in each lineage. The pseudo-time trajectories are represented by the continuous, branching lineage structures in low-dimensional data.

# Supplementary Discussion

This study revealed the key role of highly inflammatory IL1β+ neutrophils in CARDS, which are enriched in BALF and associated with progression in severe CARDS patients. Further analysis revealed that lipid metabolism reprogramming occurs within these highly inflammatory neutrophils, while extracellular macrophages can promote elevated levels of inflammation within neutrophils by releasing IL-6. In summary, the combined action of intracellular metabolism reprogramming and extracellular microenvironments shapes the highly inflammatory state of neutrophils in CARDS, which further leads to rapid exacerbation of lung injury and ARDS progression.

Firstly, we found that alveolar IL1β+ neutrophils are associated with CARDS progression. Similar to previous research, our study emphasized that neutrophils play an important role in lung injury and are associated with ARDS progression ^20,21^. More importantly, our study provides direct evidence that neutrophils, which play a crucial role in CARDS lung injury, are mainly the IL1β+ subgroup. Previous studies have reported that macrophages are the main source of IL1β ^22–26^. However, our research suggests that neutrophils are the main source of IL1β, indicating that CARDS may have heterogeneity with other lung diseases.

IL1β is a crucial type of inflammatory factor. IL1β could lead to endothelial and coagulation diseases, resulting in widespread organ damage in severe COVID-19 ^27^. Traditionally, neutrophils are considered as limiting to eradicating pathogens. Our research further supports the significant role of neutrophils in IL1β signaling, and further supports that neutrophils with a range of immune regulatory functions. Lung neutrophils have fully functional NLRP3 inflammasomes that can release IL1β through the NLRP3 inflammasome pathway during respiratory bacterial infections ^28^.

This study identified IL1β+ neutrophils as a key pathogenic factor in CARDS lung injury, providing a theoretical basis for IL-1 blocker application in severe CARDS. Although meta-analyses have summarized that previous IL-1 blockers seem to have poor therapeutic effects on CARDS ^29^, it is noteworthy that 67% to 100% of the patients in these studies received oxygen at baseline but were not intubated ^29^, leaving the effectiveness of IL-1 blockers for severe CARDS to be further explored.

Second, we found that reprogramming of lipid metabolism is a primary mechanism driving high levels of inflammation in neutrophils. Although glycolysis is the main metabolic mode of neutrophils, recent studies have found that neutrophils undergo metabolic reprogramming during differentiation, survival, and apoptosis to adapt to local microenvironmental changes ^30^, regulating neutrophil functions. Our study found that glycolysis levels in highly inflammatory neutrophils in BALF of CARDS patients significantly decreased, but the levels of fatty acid oxidation increased, indicating the occurrence of lipid metabolism reprogramming within the cells. Human neutrophils have been shown to absorb high levels of fatty acids ^31^. Fatty acids or pyruvates that provide fuel for mitochondrial respiration can rescue the differentiation of neutrophil precursors with autophagy deficiency ^32^.

We further analyzed rate-limiting enzymes in fatty acid oxidation and found that ACSL1 levels were significantly elevated in IL1β+ neutrophils. ACSL1 converts excessive fatty acids (FA) into FA-acyl-CoA esters, which can participate in various lipid metabolism pathways ^33^. Research on pseudomonas aeruginosa (PA) further verified the function of ACSL1. In PA-induced inflammation, ACSL1 promotes the formation of intracellular fatty acid crystals, which in turn activate NLRP3 bodies through lysosomal damage, promoting the synthesis and release of IL1β, resulting in the pro-inflammatory responses of PA in vascular endothelium ^34,35^. Transcriptomic research of sepsis also suggests that ACSL1 abundance in peripheral blood is correlated with the activation of neutrophil inflammasome pathways and ARDS progression ^36^. Our results suggest that ACSL1 in neutrophils could be a potential intervention target for CARDS treatment.

Third, we also found that the interaction between macrophages and neutrophils exacerbates high inflammation within neutrophils. Macrophages act on neutrophils by releasing IL-6, mediating intracellular IL1β synthesis and release. Previous studies have shown that transgenic mice overexpressing IL-6 in the lungs exhibit excessive recruitment of neutrophils into the lungs ^37^. Fibroblasts in liver cancer tissue can activate neutrophil transcription factors NF-κB and STAT3 by releasing IL-6, leading to high intracellular inflammation ^38^. In inflammatory bowel disease, functional inhibition of IL-6 leads to a decrease in neutrophils throughout the body and lungs ^39^. Therefore, the phenomenon of neutrophil hyperinflammation occurring locally in the lungs of CARDS is also a result of macrophage action.

Fourth, we also found an indirect immunosuppressive effect of neutrophils. CARDS patients in this study all experienced immunosuppression, and we further investigated the relationship between neutrophils and immunosuppression. The results showed that neutrophils can exert immunosuppressive effects on T cells through PDL1-PD1 interaction. Previous studies have also reported that tumor PD-L1+ neutrophils can negatively regulate T cells, inhibit T cell immune function, and accelerate tumor progression ^40^. In the CT26 mouse model of colorectal cancer characterized by extensive TAN infiltration, inhibition of PI3Kδ/γ enhances the efficacy of anti-PD-1 immunotherapy by inhibiting TAN immunosuppressive function ^41,42^. This also provides insights into the mechanism of immune suppression in CARDS.

This study also has limitations. Firstly, our sample size is limited. However, we set up self-control by sampling BALF from each patient before and after condition changes and conducting paired analyses. This allows us to draw reliable conclusions even with the limited sample size. In the future, we will conduct multi-center sample collection to obtain more patients meeting the inclusion criteria. Second, this article lacks rich molecular biology experimental verification. However, we have included BALF Olink proteomics as a verification for results from single-cell transcriptomics. We will also carry out further mechanism research in the future to explore the CARDS and the treatment related to IL1β+ neutrophils.

In conclusion, this study found that IL1β+ neutrophils are an important factor in the occurrence of lung injury in CARDS. The reprogramming of intracellular lipid metabolism and the pro-inflammatory effect of macrophages on them may be the initiating link of highly inflammatory neutrophils. The immune regulatory mechanism centered around neutrophils may play important roles in various ARDS. Our findings further indicate the important potential therapeutic value of IL-1 blockers and blocking inflammatory positive feedback in the alveolar microenvironment for severe ARDS.

# References

1. Dixon B, Smith RJ, Campbell DJ, et al. Nebulised heparin for patients with or at risk of acute respiratory distress syndrome: a multicentre, randomised, double-blind, placebo-controlled phase 3 trial. *Lancet Respir Med*. 2021. doi:10.1016/S2213-2600(20)30470-7

2. Korsunsky I, Millard N, Fan J, et al. Fast, sensitive and accurate integration of single-cell data with Harmony. *Nat Methods*. 2019;16(December):1-8. doi:10.1038/s41592-019-0619-0

3. Liao M, Liu Y, Yuan J, et al. Single-cell landscape of bronchoalveolar immune cells in patients with COVID-19. *Nat Med*. 2020. doi:10.1038/s41591-020-0901-9

4. Sinha S, Rosin NL, Arora R, et al. Dexamethasone modulates immature neutrophils and interferon programming in severe COVID-19. *Nat Med*. 2022. doi:10.1038/s41591-021-01576-3

5. Adams TS, Schupp JC, Poli S, et al. Single-cell RNA-seq reveals ectopic and aberrant lung-resident cell populations in idiopathic pulmonary fibrosis. *Sci Adv*. 2020. doi:10.1126/sciadv.aba1983

6. Travaglini KJ, Nabhan AN, Penland L, et al. A molecular cell atlas of the human lung from single-cell RNA sequencing. *Nature*. 2020;587(7835):619-625. doi:10.1038/s41586-020-2922-4

7. Wauters E, Van Mol P, Garg AD, et al. Discriminating mild from critical COVID-19 by innate and adaptive immune single-cell profiling of bronchoalveolar lavages. *Cell Res*. 2021. doi:10.1038/s41422-020-00455-9

8. Subramanian A, Tamayo P, Mootha VK, et al. Gene set enrichment analysis: A knowledge-based approach for interpreting genome-wide expression profiles. *Proc Natl Acad Sci U S A*. 2005. doi:10.1073/pnas.0506580102

9. Liberzon A, Birger C, Thorvaldsdóttir H, Ghandi M, Mesirov JP, Tamayo P. The Molecular Signatures Database Hallmark Gene Set Collection. *Cell Syst*. 2015. doi:10.1016/j.cels.2015.12.004

10. Wagner A, Wang C, Fessler J, et al. Metabolic modeling of single Th17 cells reveals regulators of autoimmunity. *Cell*. 2021. doi:10.1016/j.cell.2021.05.045

11. Thiele I, Swainston N, Fleming RMT, et al. A community-driven global reconstruction of human metabolism. *Nat Biotechnol*. 2013. doi:10.1038/nbt.2488

12. Wu Y, Yang S, Ma J, et al. Spatiotemporal Immune Landscape of Colorectal Cancer Liver Metastasis at Single-Cell Level. *Cancer Discov*. 2022. doi:10.1158/2159-8290.CD-21-0316

13. Aibar S, González-Blas CB, Moerman T, et al. SCENIC: Single-cell regulatory network inference and clustering. *Nat Methods*. 2017;14(11):1083-1086. doi:10.1038/nmeth.4463

14. Armingol E, Baghdassarian HM, Lewis NE. The diversification of methods for studying cell–cell interactions and communication. *Nat Rev Genet*. 2024. doi:10.1038/s41576-023-00685-8

15. Browaeys R, Saelens W, Saeys Y. NicheNet: modeling intercellular communication by linking ligands to target genes. *Nat Methods*. 2020. doi:10.1038/s41592-019-0667-5

16. Jin S, Guerrero-Juarez CF, Zhang L, et al. Inference and analysis of cell-cell communication using CellChat. *Nat Commun*. 2021. doi:10.1038/s41467-021-21246-9

17. Li H, Ma T, Hao M, et al. Decoding functional cell–cell communication events by multi-view graph learning on spatial transcriptomics. *Brief Bioinform*. 2023. doi:10.1093/bib/bbad359

18. Efremova M, Vento-Tormo M, Teichmann SA, Vento-Tormo R. CellPhoneDB: inferring cell–cell communication from combined expression of multi-subunit ligand–receptor complexes. *Nat Protoc*. 2020. doi:10.1038/s41596-020-0292-x

19. Street K, Risso D, Fletcher RB, et al. Slingshot: Cell lineage and pseudotime inference for single-cell transcriptomics. *BMC Genomics*. 2018;19(1). doi:10.1186/s12864-018-4772-0

20. Zemans RL, Colgan SP, Downey GP. Transepithelial migration of neutrophils: Mechanisms and implications for acute lung injury. *Am J Respir Cell Mol Biol*. 2009. doi:10.1165/rcmb.2008-0348TR

21. Smedly LA, Tonnesen MG, Sandhaus RA, et al. Neutrophil-mediated injury to endothelial cells. Enhancement by endotoxin and essential role of neutrophil elastase. *J Clin Invest*. 1986. doi:10.1172/JCI112426

22. Duque GA, Descoteaux A. Macrophage cytokines: Involvement in immunity and infectious diseases. *Front Immunol*. 2014. doi:10.3389/fimmu.2014.00491

23. Su Y, Chen D, Yuan D, et al. Multi-Omics Resolves a Sharp Disease-State Shift between Mild and Moderate COVID-19. *Cell*. 2020. doi:10.1016/j.cell.2020.10.037

24. Wendisch D, Dietrich O, Mari T, et al. SARS-CoV-2 infection triggers profibrotic macrophage responses and lung fibrosis. *Cell*. 2021. doi:10.1016/j.cell.2021.11.033

25. Schulte-Schrepping J, Reusch N, Paclik D, et al. Severe COVID-19 Is Marked by a Dysregulated Myeloid Cell Compartment. *Cell*. 2020. doi:10.1016/j.cell.2020.08.001

26. Schultheiß C, Willscher E, Paschold L, et al. The IL-1β, IL-6, and TNF cytokine triad is associated with post-acute sequelae of COVID-19. *Cell Reports Med*. 2022. doi:10.1016/j.xcrm.2022.100663

27. Meduri GU, Headley S, Kohler G, et al. Persistent Elevation of Inflammatory Cytokines Predicts a Poor Outcome in ARDS. *Chest*. 1995. doi:10.1378/chest.107.4.1062

28. Hassane M, Demon D, Soulard D, et al. Neutrophilic NLRP3 inflammasome-dependent IL-1β secretion regulates the γδ T17 cell response in respiratory bacterial infections. *Mucosal Immunol*. 2017. doi:10.1038/mi.2016.113

29. Davidson M, Menon S, Chaimani A, et al. Interleukin-1 blocking agents for treating COVID-19. *Cochrane Database Syst Rev*. 2022. doi:10.1002/14651858.CD015308

30. Richer BC, Salei N, Laskay T, Seeger K. Changes in Neutrophil Metabolism upon Activation and Aging. *Inflammation*. 2018. doi:10.1007/s10753-017-0725-z

31. Burns CP, Welshman IR, Spector AA. Differences in free fatty acid and glucose metabolism of human blood neutrophils and lymphocytes. *Blood*. 1976. doi:10.1182/blood.v47.3.431.431

32. Riffelmacher T, Clarke A, Richter FC, et al. Autophagy-Dependent Generation of Free Fatty Acids Is Critical for Normal Neutrophil Differentiation. *Immunity*. 2017. doi:10.1016/j.immuni.2017.08.005

33. Ellis JM, Frahm JL, Li LO, Coleman RA. Acyl-coenzyme A synthetases in metabolic control. *Curr Opin Lipidol*. 2010. doi:10.1097/MOL.0b013e32833884bb

34. Kalugotla G, He L, Weber KJ, et al. Frontline Science: Acyl-CoA synthetase 1 exacerbates lipotoxic inflammasome activation in primary macrophages. *J Leukoc Biol*. 2019. doi:10.1002/JLB.3HI0219-045RR

35. Ren G, Bhatnagar S, Hahn DJ, Kim JA. Long-chain acyl-CoA synthetase-1 mediates the palmitic acid-induced inflammatory response in human aortic endothelial cells. *Am J Physiol - Endocrinol Metab*. 2020. doi:10.1152/AJPENDO.00117.2020

36. Roelands J, Garand M, Hinchcliff E, et al. Long-Chain Acyl-CoA Synthetase 1 Role in Sepsis and Immunity: Perspectives From a Parallel Review of Public Transcriptome Datasets and of the Literature. *Front Immunol*. 2019. doi:10.3389/fimmu.2019.02410

37. Florentin J, Zhao J, Tai YY, et al. Interleukin-6 mediates neutrophil mobilization from bone marrow in pulmonary hypertension. *Cell Mol Immunol*. 2021. doi:10.1038/s41423-020-00608-1

38. Cheng Y, Li H, Deng Y, et al. Cancer-associated fibroblasts induce PDL1+ neutrophils through the IL6-STAT3 pathway that foster immune suppression in hepatocellular carcinoma. *Cell Death Dis*. 2018. doi:10.1038/s41419-018-0458-4

39. Mateer SW, Mathe A, Bruce J, et al. IL-6 Drives Neutrophil-Mediated Pulmonary Inflammation Associated with Bacteremia in Murine Models of Colitis. *Am J Pathol*. 2018. doi:10.1016/j.ajpath.2018.03.016

40. Xu P, Zhang X, Chen K, et al. Tumor Cell-Derived Microparticles Induced by Methotrexate Augment T-cell Antitumor Responses by Downregulating Expression of PD-1 in Neutrophils. *Cancer Immunol Res*. 2023. doi:10.1158/2326-6066.CIR-22-0595

41. Lin HY, Wu YZ, Chen JP, Huang SR, Wang YQ. (−)-4-O-(4-O-β-D-glucopyranosylcaffeoyl) Quinic Acid Inhibits the Function of Myeloid-Derived Suppressor Cells to Enhance the Efficacy of Anti-PD1 against Colon Cancer. *Pharm Res*. 2018. doi:10.1007/s11095-018-2459-5

42. Faget J, Peters S, Quantin X, Meylan E, Bonnefoy N. Neutrophils in the era of immune checkpoint blockade. *J Immunother Cancer*. 2021. doi:10.1136/jitc-2020-002242
